# Two-Dimensional Semiconductors for State-of-the-Art Complementary Field-Effect Transistors and Integrated Circuits

**DOI:** 10.3390/nano14171408

**Published:** 2024-08-28

**Authors:** Meng Liang, Han Yan, Nasrullah Wazir, Changjian Zhou, Zichao Ma

**Affiliations:** School of Microelectronics, South China University of Technology, Guangzhou 511442, China

**Keywords:** two-dimensional semiconductors, wafer-scale preparation, field-effect transistors, contact resistance, high-κ dielectric, post-Moore electronics, heterogeneous integration

## Abstract

As the trajectory of transistor scaling defined by Moore’s law encounters challenges, the paradigm of ever-evolving integrated circuit technology shifts to explore unconventional materials and architectures to sustain progress. Two-dimensional (2D) semiconductors, characterized by their atomic-scale thickness and exceptional electronic properties, have emerged as a beacon of promise in this quest for the continued advancement of field-effect transistor (FET) technology. The energy-efficient complementary circuit integration necessitates strategic engineering of both n-channel and p-channel 2D FETs to achieve symmetrical high performance. This intricate process mandates the realization of demanding device characteristics, including low contact resistance, precisely controlled doping schemes, high mobility, and seamless incorporation of high- *κ* dielectrics. Furthermore, the uniform growth of wafer-scale 2D film is imperative to mitigate defect density, minimize device-to-device variation, and establish pristine interfaces within the integrated circuits. This review examines the latest breakthroughs with a focus on the preparation of 2D channel materials and device engineering in advanced FET structures. It also extensively summarizes critical aspects such as the scalability and compatibility of 2D FET devices with existing manufacturing technologies, elucidating the synergistic relationships crucial for realizing efficient and high-performance 2D FETs. These findings extend to potential integrated circuit applications in diverse functionalities.

## 1. Introduction

Semiconductor technology evolve relentlessly in pursuit of smaller and faster devices with higher energy efficiency through successive generations. However, as silicon-based transistors approach their physical limits, the search for alternative materials and novel device architectures has intensified [1,2]. Among the most promising candidates, two-dimensional (2D) materials have emerged to offer a revolutionary approach to addressing the challenges of transistor scaling and advancing beyond Moore’s law [3,4,5,6].

The first discovery of graphene, which shows exceptional high mobility and mechanical flexibility in a single layer of atoms, has ignited interest in exploring 2D materials for semiconductor technology and marked a paradigm shift in transistor and integrated circuit technology [7]. Ever since, 2D transition-metal dichalcogenides (TMDC) such as MoS_2_ and WSe_2_, black phosphorus (BP), and InSe, among other 2D semiconductors with a tunable bandgap, have emerged to exhibit diverse semiconductor properties [8,9,10,11]. The extraordinary high mobility in the ultrathin 2D layers not only facilitates superior electrostatic control but also reduces short-channel effects and enhances device performance including switching speed and power efficiency [12,13,14,15]. Moreover, the ability to stack different 2D layers atom-by-atom opens up possibilities for building complex heterostructures with tailored electronic properties, paving the way for multifunctional devices and heterogeneous integrated circuits beyond conventional silicon technology [16,17,18,19,20].

**Figure 1:**
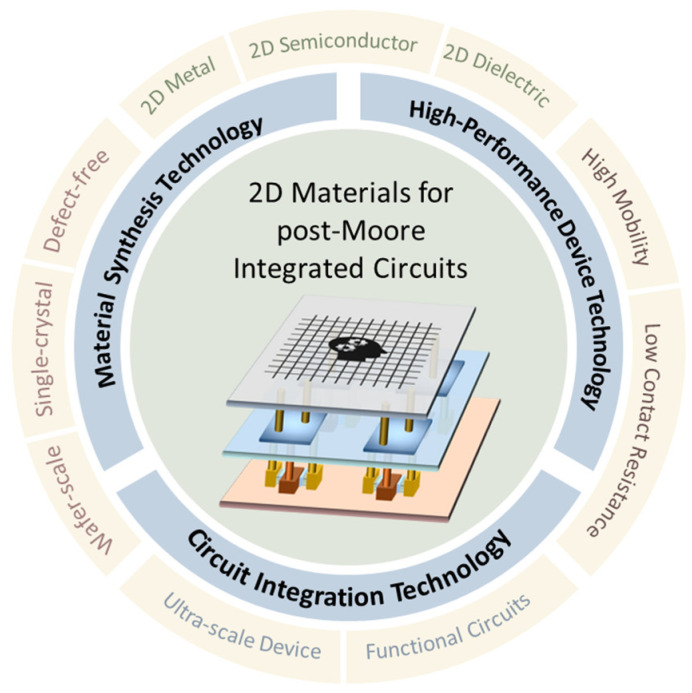
Two-dimensional materials for the post-Moore transistor and integrated circuit technology.

The use of 2D semiconductors in transistors pushes the boundaries of device miniaturization. Emerging gate-all-around FETs (GAAFETs) and complementary FETs (CFETs) exploit the unique properties of 2D materials to achieve unprecedented performance milestones in the international roadmap for device and systems (IRDS) [21,22,23,24,25]. It also encompasses a reimagining of field-effect transistor (FET) design paradigms, as various transistor architecture leverage 2D materials for enhanced scalability and to address compatibility problems [26,27,28]. As shown in Figure 1, the realization of the 2D material-based integrated circuits envisioned by IRDS necessitates large-area and defect-free 2D film growth technology, high-performance device manufacturing technology, and large-scale circuit design and integration technology.

Techniques such as chemical vapor deposition (CVD) and metal–organic chemical vapor deposition (MOCVD) have facilitated the wafer-scale growth of monolayer 2D TMDC with excellent control over the crystallinity [29,30]. These advancements are bridging the gap between prototype devices and industrial integrated circuits. Concurrently, doping strategies and defect control techniques offer versatile approaches to manifest the electronic performance of 2D devices, providing a feasible process to integrate logic circuits, driver circuits, and compute-in-memory circuits [28,31,32].

The complementary integration of 2D material-based FETs (2D FETs) into large-scale functional circuits is highly desirable. However, the fabrication of high-performance n-channel and p-channel 2D FETs remains challenging. While several works have leveraged the layered structure of 2D materials and van der Waals (vdW) assembly techniques to demonstrate high performance n-channel MoS_2_ FET prototype devices with ultralow contact resistance, the fabrication of low-resistance p-type contacts remains difficult [33,34]. As a result, p-channel silicon (Si) and WSe_2_ FETs are considered to pair with n-channel MoS_2_ FETs for complementary circuit integration [26,35]. Meanwhile, several high-κ 2D insulators have been discovered as gate dielectric in recent years, providing a clean interface and promoting high carrier mobility [36,37].

**Figure 2:**
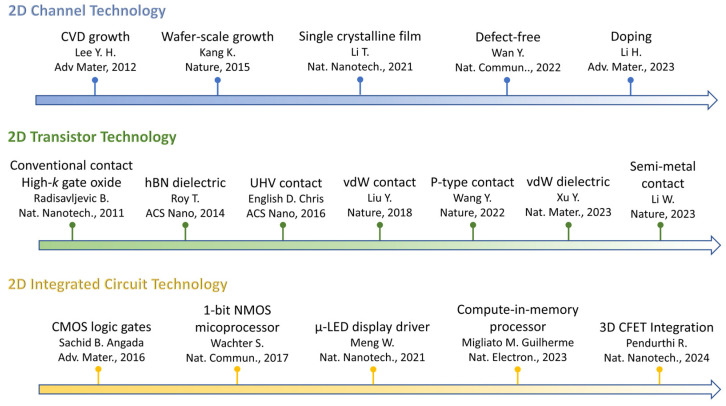
A chronological diagram showing the progress of 2D semiconductor channel technology [38,39,40,41,42], transistor technology [8,18,33,34,36,43,44], and circuit technology [28,31,32,45,46].

This review comprehensively discusses the challenges and recent advancements of 2D semiconductor technology, encompassing the spectrum from material preparation to large-scale circuit integration. Several milestones in the roadmap of 2D material preparation, transistor fabrication, and circuit integration are summarized in Figure 2. By consolidating insights from recent experimental studies, theoretical models, and technological innovations, this review highlights state-of-the-art 2D material synthesis technologies, novel transistor structures, device performance breakthroughs, and advancements in large-scale circuit integration. With an updated understanding of 2D semiconductor technology, this review addresses the remaining challenges and potential solutions suggested by recent technological advances in high-quality 2D channel preparation, high-performance 2D FETs, and monolithic three-dimensional integrated circuits.

## 2. Two-Dimensional Semiconductor Materials for Transistor Technology

High mobility and a large bandgap are essential to achieve high-speed and low-leakage FET characteristics. For most 2D semiconductors, their band structures, tunable by the layer thickness and atomic compositions, affects electronic and optical properties, such as carrier mobility, density of states, and absorption. Consequently, the selection of 2D semiconductors and their combination heterostructures becomes crucial for designing devices with specific performance requirements.

**Figure 3:**
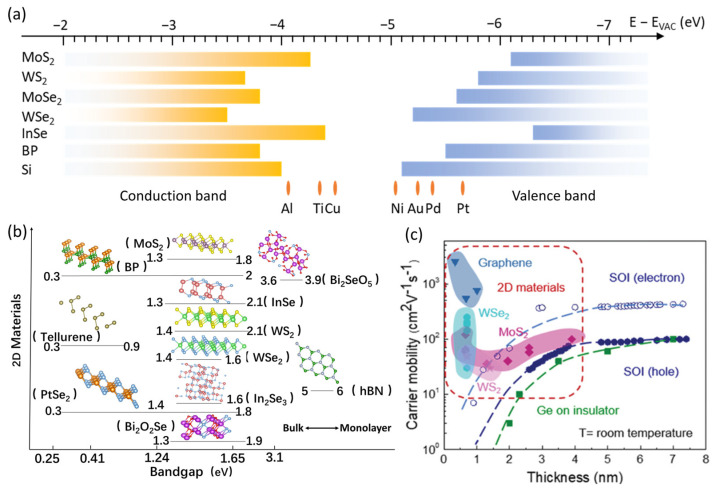
(**a**) Band alignment of 2D semiconductor monolayers compared to Si and the Fermi level of common elemental metals. (**b**) Bandgap of 2D semiconductor materials; (**c**) Carrier mobility as the thickness of the channel semiconductor film reduces to below 1 nm at room temperature. Compared with silicon and germanium, 2D materials show high mobility at the sub-nanometer scale (Reproduced with permission from [5], John Wiley and Sons, 2022).

Understanding the basic band alignment is essential when designing 2D transistors to achieve a specific polarity and low contact resistance. The band alignment of several of the most reported 2D semiconductor materials, such as Si, and popular elemental metals have been summarized in Figure 3a, respectively. For instance, a single layer of MoS_2_ has a hexagonal lattice, with its conduction band’s bottom and valence band’s top located at about 4.3 eV and 6.1 eV below the vacuum level, respectively, naturally forming n-type contacts with most elemental metals. In contrast, WSe_2_ has its valence band’s top near 5.2 eV below the vacuum level, which tends to form p-type contacts with most metals.

Furthermore, as shown in Figure 3b, 2D semiconductors show a tunable bandgap. For example, MoS_2_ shows a direct band gap of approximately 1.8 eV for its monolayer and 1.3 eV for bulk films [47,48]. This phenomenon originates from the quantum confinement effect, and the tunable bandgap property is characteristic of most 2D semiconductors, although the intensity of such an effect can vary among different materials, depending on their interlayer coupling. For example, black phosphorus exhibits a bandgap ranging from 0.3 eV to 2.0 eV, and InSe has a bandgap that spans from 1.3 eV to 2.1 eV, both showing more intense interlayer charge exchange and correlation [49,50]. Additionally, the bandgap of 2D semiconductors can be easily tuned by constructing heterostructures, applying strain engineering, performing chemical doping, and applying external electric fields [47]. For example, supporting a MoS_2_ monolayer film with an array of pillars can induce a tensile strain of up to 2%, resulting in a bandgap widening of 0.1 eV and an increase in electron mobility, thereby offering an alternative method to design device structures for enhanced carrier transport characteristics [51]. In addition, MoS_2_ sandwiched by hBN in a van der Waals heterostructure provides isolation from disorder and scattering sources including defect and charged impurities, measuring the Hall mobility up to 34,000 cm^2^(V·s)^−1^ [19].

The key attraction of 2D semiconductors is that their high carrier mobility can surpass that of Si in ultrathin films of 1 nm thickness [5]. Figure 3c summarizes the mobility of typical 2D TMDC semiconductors in comparison with graphene, silicon (Si), and germanium (Ge) in relation to their thickness. The variations in the mobility of 2D materials indicate the influence from multiple factors, including phonon scattering and surface charge scattering [19]. In a typical transistor device with a 2D semiconductor channel and oxide gate dielectric, the channel mobility can decrease due to Coulomb scattering from charged impurities of the oxide dielectrics [52]. As a result, bilayer 2D semiconductors usually exhibit higher mobility than their monolayer counterparts [12]. Theoretical mobility in a monolayer 2D TMDC can reach more than 500 cm^2^(V∙s)^−1^, and BP and InSe show even higher mobility exceeding 1000 cm^2^(V∙s)^−1^, while fabricated devices have seldomly achieved such high performance [53]. This issue can be mitigated by using a 2D insulator, hBN for example, to sandwich the channel, or by using a thicker 2D film as the channel, where the screening effect can mitigate the scattering centers [54,55]. Additionally, heavy doping by charge transfer can also alleviate the impact of charged impurities, resulting in a higher mobility [56].

Therefore, despite the theoretically exceptional electronic properties of 2D semiconductors for future transistor technology, new fabrication technologies are required to create these 2D materials with intact lattice integrity and atomically clean surfaces. These technologies to synthesize wafer-scale synthesis of single-crystal mono-/bilayer 2D films should also ensure a low thermal budget and compatibility with large-scale integrated circuit manufacturing, which is discussed in the following section.

## 3. Preparation of Wafer-Scale 2D Semiconductor Films

The synthesis of wafer-scale monocrystal 2D films is essential for fully exploiting their potential in circuit applications. To achieve this, “top-down” and “bottom-up” approaches have been developed for preparing 2D semiconductor films. The “top-down” approach primarily involves exfoliation and transfer techniques, typically used to prepare small-sized irregularly shaped 2D monocrystal flakes. This method is straightforward and capable of yielding high-quality, high-purity 2D materials with relatively low defect density. Nevertheless, it offers limited control over the deposition position and film thickness, which are considered primarily for demonstrating prototype devices and studying physics. Alternatively, the “bottom-up” approach synthesizes 2D films from atoms and molecules using methods such as chemical vapor deposition, atomic layer deposition, and epitaxy. This approach achieves uniformity over larger areas and provides better control over material thickness and composition, thereby attracting significant attention and research from the semiconductor community. This section primarily reviews the “bottom-up” methods for producing high-quality wafer-scale 2D semiconductor films and discusses the latest research advancements in growth mechanisms and techniques.

### 3.1. Controlled Synthesis of 2D Semiconductor Films

Research over the past decade has revealed a film growth kinetic that applies to most 2D semiconductor, involving random nucleation on the substrate, lateral growth of single crystalline domains, and finally, the merging of these domains into a continuous film [57,58]. From a mechanistic point of view, the synthesis method can significantly affect the crystal growth process and in turn, the properties of the resulting 2D films. Chemical vapor deposition (CVD), atomic layer deposition (ALD), molecular beam epitaxy (MBE), pulsed laser deposition (PLD), magnetron sputtering, solution process, and self-assembly methods have all achieved large-area continuous film growth of 2D materials [30,59,60,61,62,63,64]. Among them, ALD, PLD, sputtering, solution, and self-assembly methods can synthesize polycrystalline 2D material films at low temperatures below 450 °C. This offers the advantage of a low thermal budget and compatibility with the back-end-of-line (BEOL) process. However, the synthesized single-crystal 2D domain is smaller and precise control of the crystal orientation and film thickness uniformity of the domain remains challenging. As a result, these methods are usually applied to the preparation of 2D materials for the demonstration of multifunction devices but show low mobility and poor uniformity in FET devices. In contrast, CVD methods, including tubular CVD and metal–organic chemical vapor deposition (MOCVD), have been widely developed to prepare wafer-sized single-crystalline monolayer and bilayer 2D TMDC films, especially MoS_2_, whose uniformity on 12-inch wafers has been demonstrated [29,40,65,66,67]. This section focuses on the latest progress in the CVD synthesis of MoS_2_ and other 2D TMDC films for large-scale circuit integration.

Chemical vapor deposition (CVD) involves the deposition of vaporized reactants onto a substrate to synthesize 2D material thin films. Compared to traditional tubular CVD, MOCVD employs gas source pulsing, which enhances the uniformity of large-area film deposition. Herein, a series of wafer-scale semiconducting 2D films have been synthesized by stitching together randomly oriented 2D islands. For example, Wang et al. reported a multisource design in tubular CVD to achieve the epitaxy of monolayer MoS_2_ with highly oriented large domains that uniformly distributed on sapphire, with an average domain size greater than 100 μm [30]. Seol et al. synthesized 6-inch MoS_2_ and WS_2_ monolayer films on quartz glass using a pulsed MOCVD method, where the periodic precursors supply contributed to the perfect control of film thickness and uniformity [68]. Interestingly, using a designed face-to-face metal precursor supply, a 6-inch monolayer MoS_2_ film was synthesized on soda-lime glass within eight minutes, with sodium catalysts in the substrate facilitating the fast growth of monolayer MoS_2_. The salt-assisted 2D film growth and its underlying mechanism have been actively explored, elucidating an effective way to reduce the CVD synthesis temperature by lowering the activation energy [69,70].

**Figure 4:**
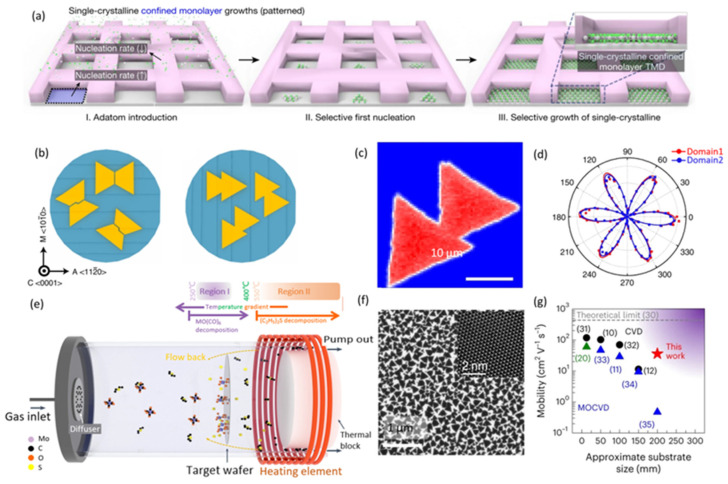
Progress of wafer-scale synthesis of 2D semiconductors film. (**a**) Schematic of the selective single-domain synthesis strategy to address the limitations of conventional TMD growth. (Reproduced with permission from [78], Springer Nature, 2023.) (**b**) The step orientations on C/M and C/A sapphire (0001) wafers and the alignment of epitaxial MoS_2_ domains corresponding to these orientations. (**c**) Mapping of polarized second-harmonic generation (SHG) from two MoS_2_ domains merging on the C/A substrate. (**d**) SHG intensity polar plot with theoretical fittings for the two domains (reproduced with permission from [40], Springer Nature, 2021). (**e**) A diagram illustrating the MOCVD system and the deposition procedure. Precursors are introduced from the left side of the reaction chamber. The molybdenum precursor begins to decompose upon reaching the left side of Region I (250 °C). The sulfur precursor decomposes in Region II (temperatures above 550 °C) and then flows back into Region I to react with the decomposed molybdenum precursor, leading to MoS_2_ deposition on the target wafer. (**f**) Scanning electron microscope image of MoS_2_ synthesized after 30 min. The inset shows a high-resolution scanning transmission electron microscope image of the synthesized MoS_2_ domains, indicating no defect sites. (**g**) A benchmark comparison of the state-of-the-art electron mobility and the size of as-grown MoS_2_ films. The purple area represents the desired range for wafer-scale MoS_2_ synthesis. Green and red symbols indicate growth temperatures below 400 °C (reproduced with permission from [29], Springer Nature, 2023).

Although great efforts have been devoted to synthesizing large-area 2D semiconducting films, atomic-resolution scanning transmission electron microscopy has revealed the polycrystalline nature of synthesized films and that the grain boundaries could degrade device performance [68]. The nucleation control at the initial growth stage plays a pivotal role in tuning the domain size and crystallinity of 2D materials [71]. Notably, the stitching of random-orientation 2D islands results in undesired grain boundaries, and thus recent research works have developed primarily two methods for CVD synthesizing large-area 2D single-crystal films. One approach involves the growth of ultra-large single-crystal domains, requiring the formation of sparse nucleation sites on the substrate surface and the adsorption of reactants at the film edges, which facilitates rapid lateral growth of the 2D film. Efforts to reduce nucleation density and eliminate grain boundaries include introducing functional groups on the film surface to suppress excessive nucleation and fabricating defects on the substrates as specific nucleation sites to synthesize large-area 2D nanosheet arrays [72,73]. These developments have led to the successful synthesis of various 2D TMDC single-crystal films in triangular shapes with sizes of several hundred micrometers and even a few millimeters [74,75,76,77]. From another perspective, Kim et al. reported a deterministic and confined growth method to synthesize monolayer WSe_2_ domain arrays, as shown in Figure 4a [78]. The nuclei were geometrically confined by patterned SiO_2_ masks on substrates, and the WSe_2_ monocrystal domains were selective grown by filling the trenches before the second set of nuclei was introduced. This method not only overcame the difficulty of growing large-area single-crystal 2D TMDC films but also showed potential for the direct construction of various vdW heterostructures.

An alternative strategy involves controlling the crystal orientation of 2D films grown on a substrate, leading to numerous crystal nuclei growing along a single lattice direction with the assistance of the atomic step edges on the substrate surface, eventually extending and seamlessly stitching together to form a continuous film with a single crystal orientation. This surface atomic step edge-guided strategy has been demonstrated as a practical growth method for the synthesis of many single-crystalline 2D TMDC and insulating hBN films, except that this method has not yet achieved the growth of single-element 2D semiconductor films such as Te and BP [79,80]. For example, Yang et al. successfully achieved the large-area stitched growth of single-crystalline MoS_2_ films on (111) Au substrates by carefully controlling the S/Mo ratio. [81]. Although the Au step-guided epitaxial method is promising and universal for synthesizing 2D TMDC single crystal films, the unavoidable transfer process can introduce defects, contaminations, and wrinkles that degrade the device performance [82,83]. Therefore, directly synthesizing high-quality 2D TMDC single crystals on insulators is highly desirable.

The Wang group developed a step-edge guide approach to achieve single-oriented MoS_2_ films on sapphire (001) substrates, which exhibited excellent electrical performance [40,66]. As shown in Figure 4b, the miscut orientation towards the α-axis of sapphire provided excellent control over the MoS_2_ domain orientation, which facilitated the nucleation and evolution of unidirectionally aligned monolayer MoS_2_ domains. As shown in Figure 4c,d, the unidirectional orientation of two MoS_2_ domains were verified by polarized second-harmonic generation with theoretical fittings for the two domains. Additionally, DFT calculations revealed that the presence of parallel steps lowered the sapphire surface symmetry and broke the formation energy degeneracy in favor of one direction. Recently, Wang et al. further combined the step-edge guide method with lattice matching between the 2D layer and the substrate, demonstrating 2-inch wafer-scale single-crystal TMDC films, including WS_2_, MoS_2_, MoSe_2_, and WSe_2_, on an α-Al_2_O_3_ surface [84].

Although promising results on the wafer-scale synthesis of monolayer 2D TMDC have already been reported, the BEOL integration of directly grown 2D materials on complementary metal–oxide semiconductor (CMOS) circuits remain challenging due to the high thermal budget required, which far exceeds the limits of silicon BEOL integration (<400 °C). To address this problem, Zhu et al. reported a dual-temperature MOCVD approach to synthesize monolayer MoS_2_ films below 300 °C, which enabled the 2D semiconductors to be synthesized directly on Si CMOS circuits without any transfer process [29]. Figure 4e illustrates the MOCVD system and the deposition procedure, showing that the sample substrate is placed in a separated low-temperature region away from the high-temperature precursor decomposition region. Figure 4f shows the scanning electron microscope image and transmission electron microscope image of MoS_2_ domains synthesized after 30 min. In Figure 4g, the obtained monolayer MoS_2_ shows a high electron mobility of ~35.9 cm^2^(V·s)^−1^ and uniformity on 12-inch wafers, representing the highest mobility achieved on industry-compatible 8- to 12-inch wafers.

### 3.2. Defect Control and Doping Techniques

Though large-area monocrystalline 2D films can be achieved by nucleation control and domain stitching approaches, atomic defects persist within these synthesized films. As a result, the obtained carrier mobilities are still far from the theoretical values due to inherent defects. Therefore, reducing surface defect density is a critical step in further enhancing the quality of 2D materials and the performance of 2D transistors.

**Figure 5:**
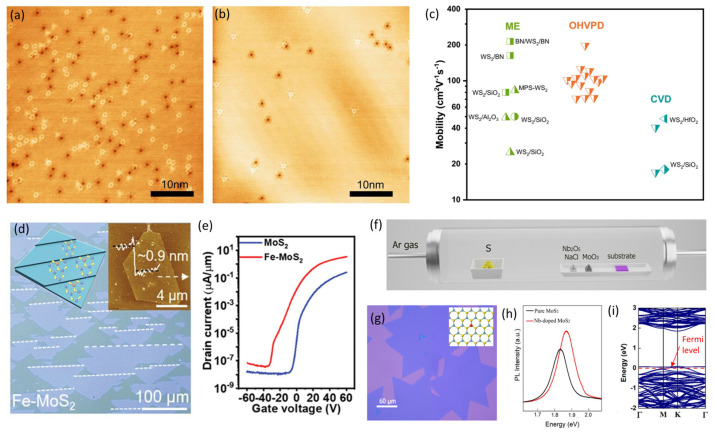
Large-area 2D semiconductor synthesis technology. STM images of a CVD-WS_2_ (**a**) and OHVPD-WS_2_ monolayer (**b**). (**c**) Comparison of mobility results for OHVPD-WS_2_, mechanically exfoliated WS_2_ monolayers, and conventional CVD-WS_2_ from the literature (reproduced with permission from [41], Springer Nature, 2022). (**d**) An optical image of Fe-doped MoS_2_ islands synthesized on c-plane sapphire, showing the unidirectional alignment of Fe-MoS_2_. Inset images are a schematic diagram of Fe-MoS_2_ on c-plane sapphire in the early growth stage (**left**), an atomic force microscope image and the height profile of monolayer Fe-doped MoS_2_ (**right**) (reproduced with permission from [42], John Wiley and Sons, 2023). (**e**) Comparison of the transfer curves of FETs using an undoped MoS_2_ channel and an Fe-doped MoS_2_ channel (reproduced with permission from [56], John Wiley and Sons, 2022). (**f**) Schematic of the CVD system for growing large-area monolayer Nb-doped MoS_2_. (**g**) An optical image of monolayer Nb-doped MoS_2_; the inset image shows its lattice structure illustrations. (**h**) PL spectra of pure MoS_2_ and Nb-doped MoS_2_. (**i**) DFT calculated band structures of Nb-doped MoS_2_ (reproduced with permission from [95], American Chemical Society, 2020).

Various atomic-scale defects in TMDC have been observed; the dominant point defects in CVD-grown TMDC have been identified as the chalcogen vacancies, with densities of the order of 10^12^–10^13^ cm^−2^ [40,85,86]. Defect-healing strategy have been developed by using alternative precursors and chalcogen monomer supply methods to promote the chalcogenization process, reducing the defect density and achieving stoichiometric TMDC films [87,88,89,90]. Particularly, Wan et al. reported that hydroxide W species as an extremely pure vapor phase metal precursor was very efficient for sulfurization, leading to about one order of magnitude lower defect density using a hydroxide vapor phase deposition (OHVPD) method compared to those from conventional CVD methods, as shown in Figure 5a,b [41]. The fabricated monolayer WS_2_ field-effect transistor (FET) reached a peak electron mobility of 200 cm^2^(V∙s)^−1^ at room temperature and 800 cm^2^(V∙s)^−1^ at 15 K, as shown in Figure 5c, comparable to those from exfoliated flakes, encouraging the industrialization of 2D materials.

Doping has been employed to control device polarity and adjust threshold voltage in Si FET technology, and this strategy has been extended to 2D semiconductor devices to realize the complementary integrated circuits. So far, n-type and p-type doping strategies of 2D semiconductors can be achieved by charge transfer, substitution, and electrostatic gating [91,92,93]. Interestingly, Li et al. developed a high-throughput CVD strategy to grow substitutional Fe-doped MoS_2_ nanosheets on SiO_2_/Si and 4-inch single crystals on c-plane sapphire, as shown in Figure 5d [42,56]. The Fe dopants resulted in heavy n-type doping and reduced the electron effective mass of monolayer MoS_2_; Figure 5e shows the improved device performance, outstanding on/off ratio of about 10^8^, and high electron mobility of about 146 cm^2^(V s)^−1^, which were even higher than those of the undoped monolayer MoS_2_. Furthermore, the contact resistance of monolayer Fe-doped MoS_2_ has been reduced to 489 Ω µm, about two orders smaller than monolayer MoS_2_ (≈117 kΩ µm).

P-type doping techniques have also been actively developed to complement advancements in n-type doping strategies. Lan et al. developed a hybrid charge transfer and molecular doping method employing self-oxidized WO_x_ to sandwich a single-layer WSe_2_ channel. A nitric oxide treatment was then applied to further enhance the p-type doping of the WSe_2_ channel, resulting in an overall effect of a record-high current of over 500 µA/µm while maintaining a high on/off ratio greater than 10^6^ [94]. Li et al. developed a substitutional Nb-doping approach to effectively tune the polarity of MoS_2_ FETs. Figure 5f shows the schematic of the one-step salt-assisted CVD method for growing monolayer Nb-doped MoS_2_. The synthesized Nb-doped MoS_2_ domain size is larger than 200 µm, and its photoluminescent spectrum shows an optical bandgap slightly smaller than the undoped MoS_2_, as shown in Figure 5g,h. Further density functional theory calculations highlight the Fermi level shifting to the valence band maximum, as shown in Figure 5i, corresponding to a p-type doping effect via Nb substitution [95]. Such a p-type doping effect has been observed in substitutional Nb-doped MoS_2_ and WSe_2_ p-channel FET devices [96,97]. These efforts are crucial for balancing the carrier type control in 2D semiconductor devices, enabling the development of complementary logic circuits.

## 4. Two-Dimensional Semiconductor-Based Complementary Transistors

Integrating complementary n-channel and p-channel 2D FETs is indispensable for constructing energy-efficient CMOS circuits. This requires that both types of FETs exhibit high performance and symmetry in threshold voltage, subthreshold slope, current drive, and power supply. Contact resistance in 2D FET is a significant factor impeding device performance. Recent advancements have optimized the contact structures and fabrication processes between metal electrodes and 2D material channels, achieving ultra-low contact resistance and notable performance improvements in n-channel 2D FETs. However, achieving low-resistance p-type contacts remains challenging, which drags back the performance of p-channel FETs and hampers the progress of 2D circuit integration. Additionally, while hBN was considered an ideal gate dielectric for 2D FETs, its relatively low dielectric constant (κ) degrades gate control and leakage performance. Recent research has introduced high-κ 2D insulating films that form atomically smooth interfaces with the channel, resulting in 2D FETs with low gate leakage, steep subthreshold swings, and high mobility. This section summarizes the advancements in high-performance 2D FETs and discusses the novel device structures for achieving low-resistance ohmic contacts, emerging high-κ 2D dielectric materials, and device integration methods.

### 4.1. Towards Low-Resistance Ohmic Contact

The large contact resistance for the metal–2D semiconductor interface is a bottleneck that limits the performance of 2D FETs. This issue is attributed to the Fermi level (E_F_) pinning effect by charge transfer mechanisms at the contact interface. This effect causes the E_F_ of a metal electrode to align with a charge-neutral level within the band gap of the 2D semiconductor, leading to the formation of Schottky contacts with high barriers [98,99]. It becomes evident that the underlying causes of Fermi level pinning are multifaceted, and the metal deposition process condition and the defect density of the 2D film could significantly modify the Schottky barrier of a metal–2D semiconductor contact and result in variable contact performance [44,100,101].

The van der Waals (vdW) contact, proposed by Liu et al. [18], forms atomically smooth interfaces between metals and 2D materials to achieve ohmic contact and reduce the contact resistance of 2D FETs. It involves transferring pre-patterned metal electrodes onto the surface of the 2D films to create vdW contacts for FET devices. As a result, the 2D channel is unaffected by defects or lattice disruption that arise from high-temperature deposition processes. Furthermore, an atomic-scale gap can form between metal atoms and 2D material atoms, isolating electron exchange between the metal and the 2D material, which helps maintain the metal’s work function and achieve ideal band alignment with the 2D material. Based on this principle, many research groups have fabricated both n-channel and p-channel FET devices with monolayer MoS_2_ and WSe_2_ active layers, using a metal work function to control the contact barrier polarity close to the Schottky Mott’s law [100,102,103,104]. Additionally, utilizing a thermally decomposable polymer as the buffer layer has enabled the integration of most CMOS compatible metals as vdW contacts for 2D FETs at the wafer scale, greatly expand the available choice of contact metals for process integration.

**Figure 6:**
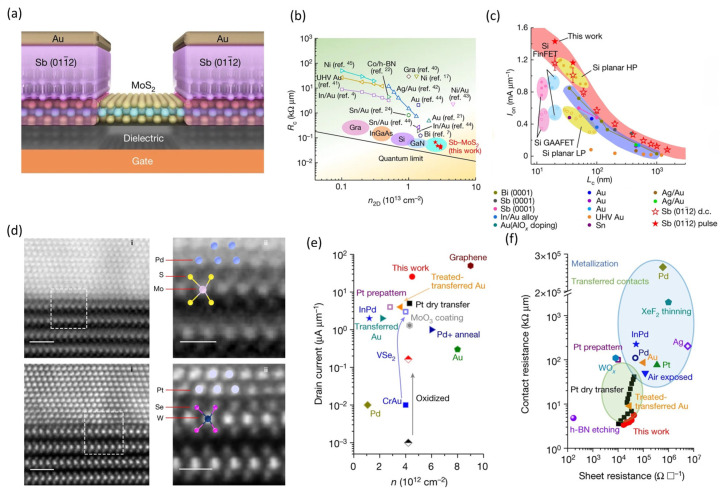
Engineering of metal–2D semiconductor contacts and benchmark of high-performance n-channel and p-channel 2D FETs. (**a**) Illustration of the monolayer MoS_2_ FET structure with semi-metallic Sb ($01\bar{1}2)$. (**b**) Contact resistance (R_C_) as a function of electron carrier concentration (n_2D_) for 2D semiconductor FETs from the literature. The black solid line indicates the quantum limit for R_C_. (**c**) Comparison of the on-state current at V_ds_ = 1 V of monolayer MoS_2_ FETs and Si planar FET, FinFET, GAAFET devices from the literature (reproduced with permission from [33], Springer Nature, 2023). (**d**) Atomic resolution ADF STEM images of Pd on multilayer MoS_2_, Pt on multilayer WSe_2_ (scale bar: 1 nm), and enlarged ADF STEM image with visible MoS_2_, WSe_2_, Pd and Pt atoms (scale bars: 5 Å). Comparison of drain current (**e**) and contact resistance (**f**) of WSe_2_ FETs with values reported in the literature using different methods (reproduced with permission from [34], Springer Nature, 2022).

Recently, metals with low melting temperatures, such as Bi, Sn, In, and Sb, have demonstrated record low contact resistance in 2D TMDC-based FETs [33,105,106,107]. The semi-metallic nature of these metals, along with their reduced damage to 2D channels during metallization, helps suppress bond association and defect generation, thereby enhancing the carrier injection of FET devices. In particular, Li et al. pushed the electrical contact of monolayer MoS_2_ close to the quantum limit by the hybridization of its energy bands with semi-metallic Sb ($01\bar{1}2)$ [33]. Figure 6a,b shows that the semi-metallic contact enhanced band hybridization and Bader charge transfer while decreasing the tunnel barrier width, resulting in a contact resistance as low as 42 Ω·µm and excellent stability at 125 °C. The improved contacts enabled short-channel MoS_2_ FETs to achieve current saturation at a 1 V drain with an on-state current of 1.23 mA/µm, an on/off ratio exceeding 10^8^, and an intrinsic delay of 74 femtoseconds, as shown in Figure 6c, outperforming equivalent Si CMOS technologies and meeting the IRDS target for 2028. However, integrating these semimetals in the BEOL process requires careful design due to the low melting temperatures (Bi: 271.4 °C, Sn: 231.9 °C, In: 156.6 °C, Sb: 630.6 °C) and their diffusive nature, which raises concerns about reliability and stability.

While achieving low-resistance n-type contacts on 2D semiconductor films using vdW contacts and low-work-function semimetals is possible, obtaining high-performance p-type contacts remains challenging. The valence band levels of 2D semiconductors, especially their monolayers, are situated around 5~6 eV below the vacuum level, which is lower than the Fermi level of most elemental metals. Consequently, forming low-barrier p-type contacts using high-work-function metals, such as platinum (Pt), palladium (Pd), and gold (Au), by conventional evaporation processes on 2D semiconductors is difficult. To address this, Wang et al. reported that maintaining device temperatures close to room temperature during the metal deposition process could minimize interface damage. Figure 6d shows the atomic resolution imaging and spectroscopy that used to verify the near-ideal clean interfaces between the 2D TMDC layers and metal electrodes [34]. Figure 6e,f shows the electrical measurements of the p-channel WSe_2_ FETs, revealing that the Fermi level is unpinned and exhibits a low contact resistance of 3.3 kΩ µm, high mobility of approximately 190 cm^2^(V∙s)^−1^ at room temperature, and a high drain current of around 30 μA/μm. Additionally, methods combining Nb doping, AuCl_3_ doping, and oxygen plasma treatment also improved p-type conduction in MoS_2_ and WSe_2_ FETs [96,108,109]. Ma et al. employed a slow evaporation approach coupled with atomic layer passivation to integrate evaporated Pt contacts with Nb-doped p-channel MoS_2_ FETs, achieving a record-high hole current of 50 µA/µm at a 1 V drain voltage, an on/off ratio of around 10^6^, and hole mobility exceeding 60 cm^2^(V∙s)^−1^ [110]. However, the record-low p-type contact resistance for 2D FETs remains several orders of magnitude higher than that of n-type contacts, which hinders the integration of complementary circuits. Thus, further research is urgently needed to address this disparity.

### 4.2. High-κ Gate Dielectrics Strategies

The MOSFET theory gives a characteristic channel length (*λ*) of FETs without short channel effect as $\lambda =\sqrt{t_{s}\varepsilon_{s}EOT/\varepsilon_{SiO2}}$, where $EOT=t_{ox}\varepsilon_{SiO2}/\varepsilon_{ox}$ is the equivalent oxide thickness, *t_s_* and *t_ox_* are the thickness of the channel semiconductor and gate dielectric, respectively, and *ε_s_*, *ε_ox_*, and *ε_SiO_*_2_ are the dielectric constants of the channel semiconductor, gate dielectric, and SiO_2_, respectively. Thus, integrating ultrathin high-κ dielectrics on a monolayer 2D channel is of great significance for ultimate transistor scaling, and a larger gate capacitance also reduces the supply voltage and energy consumption. The IRDS targets require state-of-the-art FETs to have an EOT of less than 1 nm, making it imperative to develop novel high-*κ* gate dielectric materials and integration methods for 2D FETs [25]. However, the vdW surface of 2D semiconductors presents a critical challenge in integrating high-quality gate dielectrics [111]. Although hBN has been widely reported as a promising 2D insulator that can form ideal interfaces with 2D semiconductor channels and enhance device performance [43,112,113,114], its well-known shortcomings, including a low dielectric constant (*κ* = 3~4) and the high temperatures (>1000 °C) required to grow high-quality single-crystal films [80,115], make hBN less suitable as a gate dielectric for 2D FETs in future integrated circuit applications.

**Figure 7:**
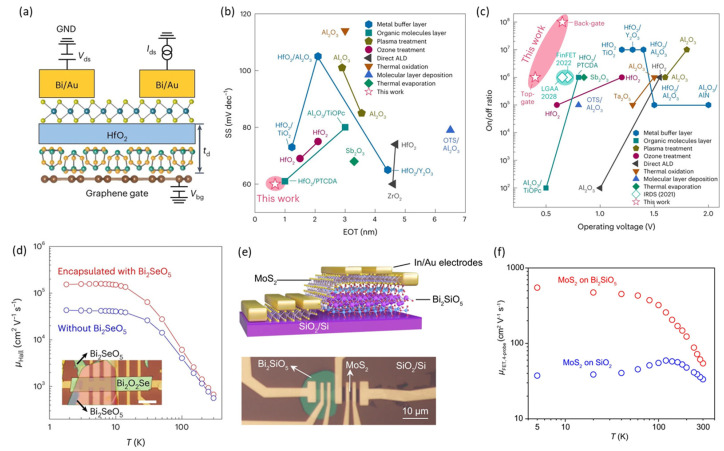
High-*κ* gate dielectrics for high performance 2D FETs. (**a**) Schematic of the MoS_2_ FET with high-κ HfO_2_ gate dielectric, an Sb_2_O_3_ buffer layer, and graphene back gates. (**b**) Comparison of the EOT and SS of hybrid Sb_2_O_3_/HfO_2_ gate dielectrics against state-of-the-art 2D FETs integrated with dielectrics via various methods. (**c**) Benchmarking operating voltages and on/off ratios of state-of-the-art 2D FETs and Si MOSFETs in the IRDS (reproduced with permission from [36], Springer Nature, 2023). (**d**) Temperature-dependent Hall mobility of 2D Bi_2_O_2_Se with and without Bi_2_SeO_5_ encapsulation, with the inset showing an optical image of the device (reproduced with permission from [120], Springer Nature, 2023). (**e**) Optical image of back-gate MoS_2_ FETs device on Bi_2_SiO_5_ and SiO_2_ substrates. (**f**) Temperature-dependent 4-probe FET mobility of MoS_2_ FETs on Bi_2_SiO_5_ and SiO_2_ substrates (reproduced with permission from [122], Springer Nature, 2023).

ALD is a mature technology for the conformal deposition of ultra-thin dielectric materials and has been predominantly used to construct high-κ gate-dielectrics in silicon transistor technologies. However, migrating the ALD technique from silicon to 2D semiconductors presents significant challenges. While ALD high-κ dielectrics can be readily deposited on CVD-grown 2D semiconductors due to surface defects, high-quality 2D semiconductors lack dangling bonds on their atomically smooth surfaces, causing ALD precursors to wet non-uniformly on the surface, leading to non-uniform and non-conformal growth of thin gate dielectrics. Although various attempts have been made to address this critical challenge, such as direct deposition [116], oxidation of deposited metal [117], surface pretreatment [118], and organic materials as a seeding layer [119], a universal approach for integrating a high-quality dielectric layer with sub-nanometer EOT on 2D materials has yet to be explored. Xu et al. demonstrated using an inorganic molecular crystal Sb_2_O_3_ as a buffer layer, as shown in Figure 7a, which could be homogeneously deposited on 2D semiconductors. This buffer layer could form a high-quality oxide-to-semiconductor interface and offered a highly hydrophilic surface, enabling the integration of high-κ dielectrics via ALD [36]. As shown in Figure 7b,c, this approach demonstrated monolayer MoS_2_ FETs with an ultrathin EOT (0.67 nm), exhibiting an on/off ratio of over 10^6^ with an ultralow operating voltage of 0.4 V, achieving high gating efficiency approaching the Boltzmann limit.

Single-crystalline gate insulators with dangling-bond-free surfaces are more competitive in fabricating high-mobility and highly reliable 2D FETs due to reduced interfacial scatterings and hysteresis. Therefore, it is highly desirable to discover new vdW insulators similar to hBN but with a much higher dielectric constant and more scalable synthetic conditions [37]. Recently, the Peng group reported the synthesis of Bi_2_SeO_5_ as a vdW high-κ gate dielectric to improve the mobility of 2D FETs [120,121]. Figure 7d shows that they fabricated 2D Bi_2_O_2_Se and 2D MoS_2_ devices with the channel encapsulated by Bi_2_SeO_5_ nanosheets. The electrical measurement results showed that the Bi_2_SeO_5_ encapsulation notably improved channel mobility, and the FETs showed a high on/off ratio > 10^8^, a low gate leakage current < 10^−6^ A cm^−2^, and a low subthreshold swing of 70 mV/dec. Furthermore, Figure 7e shows a CVD-grown Bi_2_SiO_5_ gate dielectric with an integrable fabrication process for 2D semiconductor channels demonstrated by Chen et al. [122]. The ultrathin Bi_2_SiO_5_ single crystals with thickness down to a monolayer had a high κ value > 30, a large band gap of 3.8 eV, and a large breakdown field strength. The vertically grown Bi_2_SiO_5_ had the advantage of being easily transferred onto other substrates by polymer-free mechanical pressing, which greatly facilitated its ideal vdW integration with many 2D semiconductors as high-κ gate dielectrics and screening layers. As shown in Figure 7f, the fabricated MoS_2_ FET using Bi_2_SiO_5_ as gate dielectrics showed high mobility, reaching the theoretical limit (549.3 cm^2^(V·s)^−1^). It also exhibited near-ideal gate dielectric characteristics, such as a large on/off ratio (>10^8^), an ignorable hysteresis (~3 mV), a low DIBL value (~5 mV/V), and a low gate leakage current (~10^−13^ A).

## 5. Two-Dimensional Semiconductors for Post-Moore Transistors and Circuits

As transistor sizes continue to shrink, tunneling and more short-channel effects have become increasingly apparent, and the electronic performance of FinFETs can no longer meet the demands of the next-generation integrated circuit. New transistor structures have been proposed, such as gate-all-around field-effect transistors (GAAFETs) and complementary field-effect transistors (CFETs). With their vdW layered structure, 2D materials are showing suitability and competitiveness in these novel three-dimensional FET structures and multi-tier transistor integration to extend Moore’s Law further [22,123,124,125]. Transitioning 2D FETs from the laboratory to large-scale manufacturing also involves multiple critical steps, including circuit design, process development, and large-scale production [126]. Recent advances in 2D material-based ultra-short channel FETs and milestone integration of logic gates and driver circuits have demonstrated significant progress; these technological advancements are reviewed in this section, highlighting the evolution of 2D FETs and their impact on the future of large-scale integrated circuits.

### 5.1. Two-Dimensional FETs with Extremely Short Channels

The first 1 nm gate length FET was demonstrated in a planar device form using a bilayer MoS_2_ as the channel and a single-walled carbon nanotube as the gate electrode by Hu’s and Javey’s group [12], achieving a subthreshold swing of 65 mV/dec and a high on/off ratio at room temperature. Simulations showed an effective channel length of ~3.9 nm in the off state and ~1 nm in the on state, surpassing the 5 nm limitation of Si FETs. However, directly fabricating three-dimensional (3D)-structured FETs from 2D materials remains challenging because it is difficult to grow or transfer 2D films to form vertical channels. Tan et al. addressed this issue by developing an epitaxial heterostructure using the fin-shaped Bi_2_O_2_Se channel and single-crystal high-κ Bi_2_SeO_5_ gate dielectric arranged in a vertical array [121]. These fin heterostructures achieved wafer-scale high-density growth with ultrathin fin thickness down to 1.2 nm. The fabricated 2D material-based FinFET with a 400 nm channel length exhibited high electron mobility of 270 cm^2^(V·s)^−1^, high on/off ratios of 10^8^, and a high on-state current up to 830 μA/μm. It encourages the exploration of more 2D FETs with a vertical channel to enable ultra-dense 3D integration.

**Figure 8:**
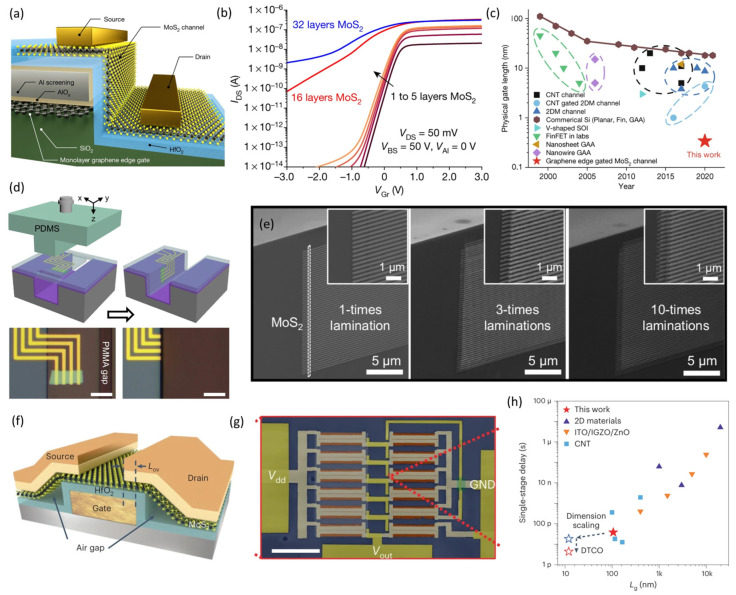
Ultra-scaled 2D FETs for the next-generation integrated circuits. (**a**) Schematic of sidewall gate structure with a monolayer MoS_2_ channel and a 0.34 nm monolayer graphene edge gate. (**b**) MoS_2_ thickness-dependent I_DS_–V_Gr_ curve in the simulated sidewall transistor. (**c**) Time scale evolution of L_g_, highlighting L_g_ scaling down to the atomic limit with CNT, 2DM, GAA, and SOI. (reproduced with permission from [23], Springer Nature, 2022). (**d**) Schematics and optical images of vertical lamination processes using a T-shape PDMS stamp laminated into the trench and the fabricated MoS_2_ vertical transistors post-lamination. (**e**) SEM images of vertical devices, with channel length and contact length fixed at 0.15 μm, channel width at 0.2 μm, source/drain electrodes at 20 nm thick Au, and an interlayer dielectric of 10 nm thick Al_2_O_3_. Insets show zoomed-in SEM images of vertical devices (reproduced with permission from [104], Springer Nature, 2024). (**f**) Schematic of the device structure of the MoS_2_ FET with air gaps. (**g**) False-colored SEM image of a MoS_2_ ring oscillator (RO) and FET structure. (**h**) Comparison of single-stage delay versus L_g_ for ROs fabricated from different semiconductor technology, with the red star indicating the measured result of the MoS_2_ RO at V_dd_ = 3.1 V (reproduced with permission from [15], Springer Nature, 2024).

Vertical field effect transistors (VFET) hold promises for developing ultra-scaled transistors. Liu et al. first demonstrated MoS_2_ vertical transistors in a gated diode structure created using a van der Waals metal integration technique [103]. The channel lengths of MoS_2_ VFETs were scaled down to 0.65 nm and 3.60 nm, achieving on/off ratios of 26 and 10^3^, respectively. To fabricate 2D FETs with gate lengths below 1 nm while maintaining a large on/off ratio, Wu et al. reported sidewall MoS_2_ transistors with an atomically thin channel and a physical gate length below 1 nm using the edge of a graphene layer as the gate electrode [23]. Figure 8a,b shows the structure and transfer characteristics of those MoS_2_ VFETs, respectively, exhibiting on/off ratios over 10^8^ and subthreshold swing values down to 117 mV/dec. Simulation results indicated that the effective length of the MoS_2_ sidewall channel approached 0.34 nm in the on-state and 4.54 nm in the off-state. As benchmarked in Figure 8c, this work demonstrated a FET with the shortest channel length ever achieved and highlighted the potential to extend Moore’s law of transistor scaling down for next-generation integrated circuits. Recently, Tao et al. developed a T-shape lamination approach for fabricating 2D VFETs with sidewall channels and 10 layers of laterally stacked 2D VFETs, as shown in Figure 8d,e. This method achieved a high device density of over 10^8^ cm^−2^, overcoming the incompatibility between planar fabrication processes and vertical device structures [104].

Vertically integrated complementary field-effect transistors (CFETs) using hetero-2D semiconductors with different carrier types have been demonstrated in recent years [13,14,26,35,45,93,127]. However, the lack of a stable and non-destructive doping scheme for 2D semiconductors has hindered the monolithic integration of complementary logic circuits. Guo et al. addressed this by placing MoS_2_ on a vdW antiferromagnetic insulator, chromium oxychloride (CrOCl), to control the carrier polarity of MoS_2_ from n-type to p-type through strong vdW interfacial coupling [128]. This approach achieved room-temperature hole mobilities up to approximately 425 cm^2^(V·s)^−1^, on/off ratios reaching 10^6^, and air-stable performance for over one year. Three-dimensional complementary logic circuits constructed using 14 vdW stacked 2D material layers, including inverters, NAND gates, and SRAM, have been demonstrated. That work highlights the potential of vdW intercalation for 2D FET polarity engineering, paving the way for robust three-dimensional integrated circuits based on 2D material-based logic gates.

Despite progress on single-transistor devices, developing high-frequency integrated circuits remains a challenge. Very seldom can current 2D material-based FETs and circuits operate in the gigahertz range [31,129,130], well below the speed achieved by Si CMOS and emerging carbon nanotube technology [131]. Fan et al. tackled this issue by developing an innovative air-gap spacer structure in monolayer MoS_2_ FETs, as illustrated in Figure 8f [15]. This device structure facilitated doping-free ohmic contacts and minimized parasitic capacitance. Figure 8g,h exhibits the fabrication of five-stage ring oscillators using MoS_2_ FETs with a 100 nm channel length, operating at 2.65 GHz (delay < 40 ps), achieved through a design-technology co-optimization (DTCO) process. Further technology computer-aided design (TCAD) simulations indicate that the air-gap structure could be scaled down to the 1 nm technology node and meet the IRDS targets for 2031. To provide a straightforward understanding for the reader, Table 1 compares the electronic properties, transistor performance, and circuit integration capabilities of Si, indium gallium zinc oxide (IGZO), carbon nanotubes (CNT), and MoS_2_ as a representative 2D semiconductor. Although circuits based on MoS_2_ achieve a much lower integration density, MoS_2_ demonstrates superior mobility and transistor performance in ultrathin channels compared to conventional semiconductor materials, highlighting its advantages in device miniaturization for the next-generation integrated circuit.

### 5.2. Functional Circuit Integration

**Figure 9:**
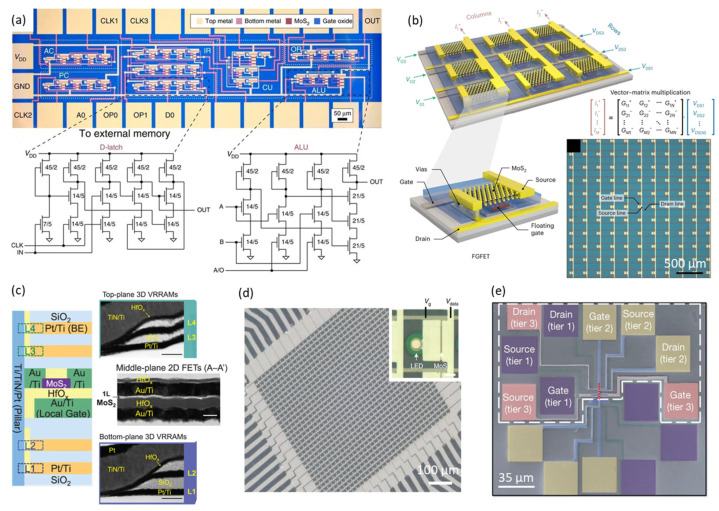
Large-scale fabricated MoS_2_ FET array and circuit integration. (**a**) Microscope image of the microprocessor and the circuit schematics of a D-Latch and an ALU, with W/L ratio in units of μm/μm for each transistor (reproduced with permission from [31], Springer Nature, 2017). (**b**) Three-dimensional rendering of MoS_2_ FGFETs in a matrix array, with gate and drain contacts organized in rows, source signal applied to columns, gate signals on the left, and drain signals on the right. The inset shows the optical image of the memory matrix and vector–matrix multiplication signal correspondence (reproduced with permission from [32], Springer Nature, 2023). (**c**) Cross-sectional structure of the 1T–4R 3D VRRAMs, with insets showing the cross-sectional transmission electron microscope images of the top-plane VRRAMs (L3 and L4), the middle-plane 2D MoS_2_ FETs, and the bottom-plane (L1 and L2); the scale bars are 50 nm (reproduced with permission from [137], Springer Nature, 2023). (**d**) Optical micrographs of the AM micro-LED display and inset showing the 1T1D pixel; the scale bar is 5 μm (reproduced with permission from [28], Springer Nature, 2021). (**e**) Top view of false-colored SEM image of a three-tier 3D device stack, with color-coded contact pads (gate, source, and drain) representing tiers 1, 2, and 3 (reproduced with permission from [125], Springer Nature, 2024).

The development of device arrays and integrated circuits using 2D MoS_2_ has seen significant progress, owing to its relatively advanced large-area synthesis and device fabrication technologies compared to other 2D materials. Wachter et al. presented for the first time an NMOS-logic 1-bit microprocessor implemented using planar MoS_2_ FETs. Figure 9a shows that the MoS_2_ microprocessor integrated with 115 transistors could execute user-defined programs stored in external memory, perform logical operations, and communicate with peripheral components, representing the most complex circuitry achieved with a 2D material to date [31]. Nevertheless, complementary logic circuits have only been demonstrated on a much smaller scale due to significant challenges in achieving performance uniformity for both n-channel and p-channel 2D FETs across large areas [13,93,128,135]. Consequently, although 2D FETs have shown superior performance compared to Si in ultra-short channel devices, their potential as replacements for Si in future electronic technologies remains largely unexamined, as direct comparisons at the circuit level are still lacking. Recently, Lu et al. addressed this gap by comparing 2D-based and Si-based static random-access memory (SRAM) circuits across technology nodes ranging from 16 nm to 1 nm [136]. They employed calibrated MoS_2_ n-channel FETs and WSe_2_ p-channel FETs and utilized TCAD for their device and circuit simulations. The findings indicated that 2D-based SRAM outperformed Si SRAM in terms of stability, operating speed, and energy efficiency. Specifically, planar 2D FETs exhibited a lower capacitance compared to three-dimensional Si FETs at the 1 nm node, resulting in a 16% reduction in cell read access time, a 72% reduction in write time, and a 60% decrease in dynamic power. These results highlight the potential of 2D FETs for counteracting performance degradation caused by the reduced metal pitch and increased wire resistance in advanced technology nodes, presenting promising prospects for high-performance and low-power circuit applications.

On the other side, the heterogeneous integration of wafer-scale 2D transistors into driver circuits for advanced computing, display, and memory technologies has been extensively explored. As shown in Figure 9b, Marega et al. reported a 32 × 32 vector–matrix multiplier integrated with 1024 floating-gate MoS_2_ FETs to process signals in artificial neural networks and extract meaningful information from the massive quantities of data produced in the real world [32]. Their wafer-scale fabrication process achieved a high yield and low device-to-device variability, which are critical for practical applications.

To further minimize latency and energy consumption in emerging data-intensive computation architectures, Xie et al. demonstrated a monolithic 3D integration of atomically thin MoS_2_ transistors with 3D vertical resistive random-access memory (VRRAM), as shown in Figure 9c [137]. Their fabrication process, conducted at temperatures below 300 °C, ensured that the top-plane fabrication did not impact the performance of bottom-plane devices. The MoS_2_ transistors enabled each VRRAM layer to be driven into four distinct resistance states (2 bit), with circuit-level modeling highlighting advantages over planar memory in terms of reduced area, faster data transfer, and lower energy consumption, underscoring its potential for energy-efficient on-chip memory systems. Additionally, as shown in Figure 9d, Meng et al. integrated high-current MoS_2_ thin-film transistors (TFTs) with nitride micro-light-emitting diodes (µLEDs) through a BEOL process, achieving an active-matrix display with a resolution of 1270 pixels per inch for augmented and virtual reality applications [28]. The MoS_2_ TFTs exhibited a drive current of 210 μA/μm and excellent uniformity, driving the µLEDs to a luminance of 7.1 × 10^7^ cd/m^2^ at low voltage, surpassing the capabilities of existing TFT technologies based on amorphous silicon (a-Si) and indium gallium zinc oxide (IGZO).

The 3D integration of circuits not only enhances device density for the “More Moore” roadmap, but also introduces multifunctional capabilities for the “More than Moore” technologies. While Si-based 3D integrated circuits are commercially available, efforts to apply 2D materials to 3D integration and develop their unique functionalities are limited. In this context, the Das group made significant strides by demonstrating several 3D integration approaches using 2D semiconductors [46,125]. They achieved several milestones, including wafer-scale integration of multi-tier MoS_2_ and WSe_2_ FETs, with over 10,000 FETs per tier, as illustrated in Figure 9e. Furthermore, they accomplished a two-tier 3D integration featuring 200 MoS_2_ FETs per tier, each with a short channel length of 45 nm. Additionally, they realized a 3D circuit that showcased multifunctional capabilities, including sensing and storage. These advancements demonstrate the potential for more complex and higher-density integrated circuits with increased monolithic layers and diverse functionalities, providing a reference for the development of 3D integrated circuits in the post-Moore era.

## 6. Conclusions

Two-dimensional semiconductors hold remarkable promise for revolutionizing future field-effect transistors and integrated circuits. Their superior electronic properties and compatibility with state-of-the-art CMOS technology position them as key enablers for advancing the performance and functional diversity of integrated circuits. Recent developments have demonstrated substantial progress and notable milestones in wafer-scale growth and high-performance device fabrication, highlighting the potential of 2D semiconductors in FET applications. Nonetheless, several challenges remain, including achieving defect-free integration, optimizing doping strategies, clean van der Waals (vdW) interfaces to contact and dielectrics, the integration of high-*κ* dielectrics, and ensuring design-fabrication optimization. Addressing these challenges requires leveraging insights from established semiconductor technologies and adapting them to the unique characteristics of 2D semiconductors. Key areas for further research include refining wafer-scale growth mechanisms, developing precise doping techniques, and enhancing interface quality to improve device performance. Solutions include developing clean interfaces through advanced deposition methods and self-passivating approaches, employing dielectric materials like hBN to preserve electronic integrity, all while ensuring compatibility with existing CMOS processes for industrial scalability. In addition, creating compatible circuit design tools and optimizing processes from material synthesis through system integration in a design technology co-optimization manner are critical to advancing circuit application of 2D semiconductors. The integration of 2D materials into state-of-the-art FETs and integrated circuits offers the potential for significant technological advancements. By overcoming existing hurdles and continuing interdisciplinary collaboration, 2D materials are set to drive innovation and redefine the capabilities of semiconductor technology in a new paradigm.

## Figures and Tables

**Table 1:** Comparison of electronic properties and transistor technology across Si, IGZO, CNT, and 2D MoS_2_.

Material	Bandgap (eV)	Mobility (cm^2^V^−1^s^−1^)	I_ON_ (μA/μm)	On/Off Ratio	Channel Length	Channel Thickness	Integration Scale	Ref.
Si	1.12	100	800	10^5^	30 nm	6 nm	10^9^ cm^−2^	[25]
IGZO	3.5	13.5	10	10^9^	0.2~10 μm	10~100 nm	442 cm^−2^	[132,133]
CNT	0.4~0.8	10^4^	10	10^5^	5 nm	1 nm	10^4^	[131,134]
MoS_2_	1.3~1.8	3	2	10^8^	2 μm	1.3 nm	115	[31]
		70	1230	10^8^	20 nm	0.65 nm	1	[33]
		/	0.69	10^5^	0.34 nm	0.65 nm	1	[23]

## Data Availability

Data are available upon request.
